# Supplementary material for: Ankyrin repeats in context with human population variation
Source: PLoS Comput Biol. 2021 Aug 24;17(8):e1009335. doi: 10.1371/journal.pcbi.1009335 (PMC8415598; doi:10.1371/journal.pcbi.1009335)
Supplement: Attachment — Submitted filename: ankyrins_reviewers_response_v4_August2021.docx [file pcbi.1009335.s001.docx]

**Response to reviewers**

**Ankyrin Repeats in Context with Human Population Variation**

We thank all three referees for their positive comments about our manuscript and helpful suggestions. We explain below how we have responded to each reviewer’s comments and how we have modified the manuscript to accommodate them. We have also made some minor changes to the text to correct small typos and language errors we noticed.

Reviewer #1:

The paper entitled “Ankyrin Repeats in Context with Human Population Variation” describes single nucleotide variation from a human population within a large ANK repeat superfamily. They interpret the variation in the context of ANK conservation and structure and suggest their methods would apply to understanding other protein families. The authors present a great deal of introductory material on known characteristics of the repeat and their data generally support the known characteristics of the fold. The novelty includes introduction of a basal surface based on RSA as well as interpreting positions enriched and depleted in missense mutations. Overall, the work is presented clearly with a few minor points elaborated below.

I think the strategy of combining all repeats into one MSA is useful in that it “boosts the statistical power” as the authors point out. Unfortunately, the use of repeats makes the method less appealing for all folds in general, and the author statement should be clarified accordingly.

Reviewer #1 makes a good point regarding statistical power. In our earlier work with 1,291 Pfam families (MacGowan *et al.*, 2017) we discovered that aggregating variants across human paralogs could identify protein-protein and protein-ligand interaction sites. Our motivation in studying repeat families such as the Ankyrin was to further boost that signal. We’ve added a few sentences in the last paragraph of the introduction, page 5, lines 129-134 to clarify and emphasise this point.

It is also interesting in that the use of repeat MSA probably removes signals from ortholog conservations, allowing insight into specificity. Perhaps more attention should be paid to the relative content of the MSA in terms of ortholog vs. paralog, vs repeat.

We agree this would be an interesting avenue to explore in more detail. However, in our experience, defining orthology and paralogy within repeat families is not straightforward. A major strength of the approach applied in this paper is that it sidesteps the need to classify the sequences as paralogs or orthologs yet identifies sites purely from sequence and population data that make sense structurally.

There are a variety of approaches that attempt to predict functionally important sites by sub-dividing MSAs on sequence similarity or structural or functional criteria. Indeed, many current methods build on ideas from our early work on this problem (Livingstone and Barton, 1993). We think there is definitely more research to be carried out in this area, but this would require analysis of a wide range of repeat families and so we feel this is beyond the scope of the current manuscript.

Finally, the examples are informative and represent the most common binding surface. However, examples of binding to the other surfaces might also be informative, especially for the newly introduced basal surface. Perhaps it may add non-specific interaction surface that is robust to mutation? Perhaps it allows for adaptive evolution?

These are interesting hypotheses suggested by Reviewer #1. We find that there are very few interactions with the basal surface, and these are dominated by interactions with single residues. This contrasts with binding on the concave surface where usually all the residues of the surface participate in the binding. Accordingly, our results suggest that the basal surface is not important for substrate binding. To elaborate more on the few examples of non-concave binding, we have modified the subsection of Results and Discussion named *“Different surfaces of the ARD”*, found in page 21, lines 487-496. We now describe the four examples of non-concave binding that we observed in our dataset.

I think the materials used for the analysis should be provided, including the MSA as well as lists of the PDBs.

There is a public GitHub repository containing the code used in our analyses as well as the main data tables that are used as input in our analysis. The multiple sequence alignment to which the work refers back and which overview is depicted in Figure 4 in the manuscript is also present in the repository, which can be found in: <https://github.com/bartongroup/JSU_ANK_analysis> , with DOI: 10.5281/zenodo.5139587.

Minor comments:

line 69 and &1 are missing commas, please check the manuscript for similar omissions.

Noted and fixed.

line 82 awkward sentence, suggested change “in this turn, explaining why ASP32 is conserved. Gly would be…”

Appreciate the suggestion and modified accordingly.

line 107 reference to “evolutionary unconserved positions” that were “enriched in ligand, DNA and protein interactions” is confusing given the idea that functional sites tend to be conserved.

We agree with Reviewer #1 in that this sentence lacked clarity in the original manuscript. We slightly modified the text in lines 119-121, page 5, to remove confusion.

Perhaps including the concept of ortholog vs. paralog conservations would be helpful.

We have modified a paragraph in the introduction to briefly introduce this concept. Found in page 5, lines 106-111.

Figure 3 please include a more detailed description of the upset plot, including what the columns and rows represent.

We have modified the legend of Figure 3 to explained with more detail what the UpSet plot represents. Hopefully, the plot can bet better understood now. Page 6, lines 151-155.

Line 215-217. Please explain how you defined meaningful biological units. For example, what are “preferred assemblies”. Do protein interactions include homodimers or are they limited to different chains?

We did not define the biological units ourselves, but took them from the PDBe, as stated in the original manuscript. We have added more detail on how the PDBe defines the biological units or preferred assemblies. This can be found in page 9, lines 251-255. Homodimers are not included in our definition of protein-protein interactions. This definition only includes interactions between different proteins, this is clarified in page 9, line 256.

Figure 6. I suppose I do not understand the difference between the colour ranges and the height of the bars. Each are described as normalized Shenkin scores? Perhaps I am confused because the green and red are flipped?

As Reviewer #1 noticed, the colour legend of Figure 6 was wrong, a fixed version can now be found in the revised manuscript. They are also right in that the height of the bars and their colour represent the same Normalised Shenkin divergence score. The aim behind this colouring scheme was to further facilitate the identification of highly conserved or divergent positions according to the thresholds we used. It is our belief that this colouring scheme facilitates the overall understanding of the conservation profile of the ankyrin repeat motif.

Also, “normalised” is spelled wrong.

Throughout the manuscript, we consistently use British spelling which favours -ise. We would be happy to change the manuscript to American spelling, should the editor wish it.

Examples of the different binding modes might be informative. Is there something unique about the bound structures for the less observed convex or combination binding modes? Perhaps the mixed mode includes whole protein binding partners, while the others might be peptides?

Yes, there is something unique indeed. There are only three structures that illustrate a binding mode that is not the absolute concave one. One of them is dominant convex and the other two show ankyrin repeat domains binding their substrates in a mixed binding mode. One of them shows how two different ankyrin repeat domains stack one after the other to form a longer super-domain. The other shows how two different ankyrin repeat proteins bind to each other involving residues from all surfaces and even buried ones. The uniqueness of these examples lies on the way these repeat domains bind their substates, instead of whether these are whole proteins or just peptides. We elaborate more on the few examples of non-concave binding in the modified subsection of Results and Discussion named *“Different surfaces of the ARD”*, found in page 21, lines 487-496.

Reviewer #2:

The authors perform a comprehensive analysis of structural and functional features of Ankyrin proteins and their variants. The analysis identifies some novel trends regarding the features of missense mutations, and particularly, it identifies five positions that do not get mutated and have distinct structural features than other residues. The authors also discuss the relevance of the results for protein modeling and engineering.

The topic studied is important, the manuscript is well written, and the analysis is carefully designed. The data and scripts are available via github. I have the following minor comments and suggestions.

While the focus of the study is the analysis of the missense mutations, this part of the study is a bit lacking. In particular, we do not know if the missense mutations analysed had any functional effect. Thus, it would be informative to further analyse the missense into likely neutral mutations, which would be common variants with no reported effect, as well as disease mutations or at least those that are predicted to have some kind of functional effect.

All the variants we analyse come from gnomAD which has exomes and genomes from unrelated individuals from control groups in control-case studies datasets. Individuals affected by paediatric diseases were removed as well as their relatives. For this reason, disease-variants might still be included in gnomAD, although at a frequency equivalent or lower than in the general population. Consequently, most of the variants found in gnomAD are expected not to be pathogenic. We also looked at ClinVar, which contains associations between genetic variants and their phenotypes and so explicitly identifies disease variants. We found very few variants thought to be disease-related and so it was not possible to draw any reliable conclusions about their location frequency or type. We have modified the manuscript on page 7-8, lines 193-198, as well as page 9, lines 242-249 to explain and clarify this aspect.

It would be interesting if the authors could comment on the overall flexibility of the Ankyrin domain, and if/how it affects the different regions and their interaction with binding partners.

We did not look into the structural flexibility or the energetics of the domain. However, we realise this is an important angle and so have added a paragraph about it in the introduction, page 4-5, lines 94-105. This is as much as we have been able to find in the literature. As far as we know, not much MD or other techniques have been used to study this property of ankyrin repeat domains.

Reviewer #3:

This is a very comprehensive study of ankyrin-repeat proteins, a ubiquitous class of proteins in nature that are also of great interest as biotherapeutics, and hence the findings are important. I recommend publication with a point of clarification requested:

One of the main findings of the sequence analysis is that there are three 'surfaces' on these proteins - in addition to the concave surface where ligand-interactions have been most frequently observed to date, there is the convex surface and also a ridge. Can the authors provide some clarification on their conclusions about the other two surfaces - are they saying that these might potentially be interaction interfaces on the basis of the observed sequence variations, and can they clarify how many of the proteins analysed are known to use these surfaces for binding?

In the previous literature, the surface of the ankyrin repeat domain is divided in two faces: concave and convex. In our work, by using the RSA we divide the classic convex surface into convex and basal. Therefore, three surfaces in total. On page 17, lines 406-415, we discuss the enrichment in missense variants on a surface level. On page 20-21, lines 467-479, we do the same for protein-protein interactions. Immediately after, in the *“Different surfaces of the ARD”* subsection, we discuss how the distribution of missense variants and protein-protein interactions at a surface level are related. We have modified the text and now discuss further the different binding modes, their observed frequencies in our dataset and the specific examples of non-concave binding that are known.
